# Iontophoresis-Based Topical Drug Delivery for Dermatologic Conditions: A Systematic Review

**DOI:** 10.3390/ph19050765

**Published:** 2026-05-13

**Authors:** Francesco Piscazzi, Francesco D’Oria, Maria Alejandra Ramirez, Marco Ardigò

**Affiliations:** 1 Dermatology Unit, IRCCS Humanitas Research Hospital, 20089 Rozzano, Italy; francesco.doria@humanitas.it (F.D.); marco.ardigo@hunimed.eu (M.A.); 2 Department of Biomedical Sciences, Humanitas University, 20072 Pieve Emanuele, Italy; 3 Degree Course in Medicine and Surgery, Humanitas University, 20072 Pieve Emanuele, Italy; maria.ramirez@st.hunimed.eu

**Keywords:** iontophoresis, transdermal drug delivery, dermatology, topical therapy, systematic review

## Abstract

**Background/Objectives**: The efficacy of topical therapies in dermatology is often limited by the barrier function of the stratum corneum, which restricts drug penetration. Iontophoresis is a non-invasive transdermal delivery technique that uses a low-intensity electrical current to enhance the transport of charged and polar molecules across the skin. It has emerged as a strategy to improve local drug bioavailability while minimizing systemic exposure. We systematically reviewed the clinical evidence on the efficacy, safety, and pharmacologic performance of iontophoresis-assisted topical drug delivery in dermatologic diseases. **Methods**: This systematic review followed PRISMA guidelines and was prospectively registered in PROSPERO (CRD420251234877). PubMed, Embase, Web of Science, CENTRAL, and ClinicalTrials.gov were searched through 19 November 2025 without language restrictions. Records were screened against predefined eligibility criteria, and data were extracted on study design, participants, dermatologic indications, intervention/comparator, iontophoresis parameters, efficacy outcomes, and adverse events. The risk of bias was assessed using RoB 2 for randomized trials and the JBI checklist for non-randomized studies. Because of substantial clinical and methodological heterogeneity, the findings were synthesized narratively and no meta-analysis was performed. **Results**: Twenty-one studies published between 1990 and 2025 met the inclusion criteria, including 15 randomized and 6 non-randomized studies. Investigated conditions included psoriasis, eczema, melasma, post-inflammatory hyperpigmentation, herpes labialis, onychomycosis, chronic ulcers, systemic sclerosis-related digital ulcers, acne scarring, and actinic keratosis. Across studies, findings were mixed. The most consistent signals of benefit were observed in pigmentary disorders and infectious diseases, whereas results were more heterogeneous in inflammatory dermatoses and some studies did not show superiority over active comparators. Tolerability was generally favorable, with adverse events limited to mild, reversible local reactions such as erythema, tingling, burning, or transient irritation. No serious treatment-related adverse events were reported. **Conclusions**: Iontophoresis may represent a useful non-invasive delivery-enhancement strategy in selected dermatologic settings, particularly when topical efficacy is limited by anatomical or physicochemical barriers. However, heterogeneity in protocols, formulations, outcomes, and clinical indications limits direct comparison and does not support broad conclusions of efficacy across all dermatologic conditions. Larger, standardized trials are needed to clarify its therapeutic role, long-term efficacy, and indication-specific benefit.

## 1. Introduction

Among the therapeutic methods for dermatological conditions, transdermal delivery of drugs is an essential component. However, the properties of the thick and protective stratum corneum significantly reduce the effective penetration of pharmacologic agents, thereby limiting their therapeutic action [1]. Consequently, many physical and chemical methods that enhance the penetration of these agents have been studied, including occlusion, microneedling, phonophoresis, lasers, and microemulsions. Each of these techniques appears to have some restrictions in terms of penetration depth, patient tolerability, reproducibility, and the possibility of disrupting the skin barrier [2].

One of the non-invasive techniques that has been studied to improve the penetration of therapeutic agents is iontophoresis, which involves the delivery of a low-intensity electric current that promotes the movement of charged or polar molecules through the skin. Theoretically, it allows for better control of drug flux and a more localized approach, which minimizes systemic exposure, particularly for drugs that are poorly absorbed through the stratum corneum (e.g., ionic therapeutics). These properties make it possible to deliver drugs that normally would be insufficiently absorbed by the skin layers, expanding the range of deliverable drugs beyond those achievable with passive topical application [3]. Over the years, many different dermatologic conditions have been treated using iontophoresis to evaluate its efficacy in enhancing the transdermal delivery of drugs. These conditions range from those with inflammatory mechanisms, such as psoriasis and eczema, to disorders of pigmentation such as melasma and post-inflammatory hyperpigmentation, to infectious diseases such as herpes labialis and onychomycosis, to ulcerative disorders such as venous ulcers and systemic sclerosis-related digital ulcers, and to acne scarring. Furthermore, it has been used in photodynamic therapy to improve the penetration of photosensitizers [4].

Although several clinical studies have investigated iontophoresis in dermatology, the available evidence remains heterogeneous in terms of study design, delivered agents, formulations, current parameters, exposure time, follow-up duration, and outcome measures [5,6]. Therefore, this systematic review aimed to evaluate the clinical efficacy, safety, and pharmacologic performance of iontophoresis-assisted topical and transdermal drug delivery across dermatologic indications, while contextualizing its role in comparison with conventional topical therapy and other penetration-enhancement approaches.

## 2. Methods

### 2.1. Study Design

This systematic review was conducted in compliance with the PRISMA (Preferred Reporting Items for Systematic Reviews and Meta-Analyses) guidelines. The protocol was prospectively registered in the PROSPERO database (registration number: CRD420251234877).

### 2.2. Inclusion and Exclusion Criteria

We adopted specific eligibility criteria to evaluate the efficacy and safety of iontophoresis in dermatology. We included randomized and non-randomized controlled trials, observational studies (cohort and case-control), and case series. The review considered studies involving human participants of any age and sex treated with iontophoresis-based topical or transdermal drug delivery for dermatologic conditions. We explicitly excluded studies focusing on hyperhidrosis or the use of iontophoresis for local anesthesia, as these applications are well-established and distinct from the therapeutic scope of this review. Furthermore, narrative reviews, systematic reviews, case reports, and conference abstracts without full text, animal studies, and in vitro studies were excluded. No language restrictions were applied.

### 2.3. Search Strategy

The literature search was designed to identify studies evaluating the pharmacologic performance and clinical outcomes of iontophoresis. Searches were conducted up to 19 November 2025 across major electronic databases: PubMed, Embase, Cochrane Central Register of Controlled Trials (CENTRAL), Web of Science, and ClinicalTrials.gov. The search was performed by two independent reviewers (F.P. and M.A.R.). Any discrepancies were resolved by a third reviewer (M.A.), after which the final search strategy was agreed upon. Search criteria combined terms related to the delivery system (e.g., “Iontophoresis”, “Transdermal”, “Electro-driven delivery”) with terms related to dermatologic indications and outcomes (e.g., “Skin diseases”, “Dermatitis”, “Drug penetration”). The full search strategies for PubMed, Embase, Web of Science, CENTRAL, and ClinicalTrials.gov, including all search terms, Boolean operators, and any applied limits, are provided in Supplementary Table S1.

### 2.4. Data Extraction and Study Selection

**Figure 1:**
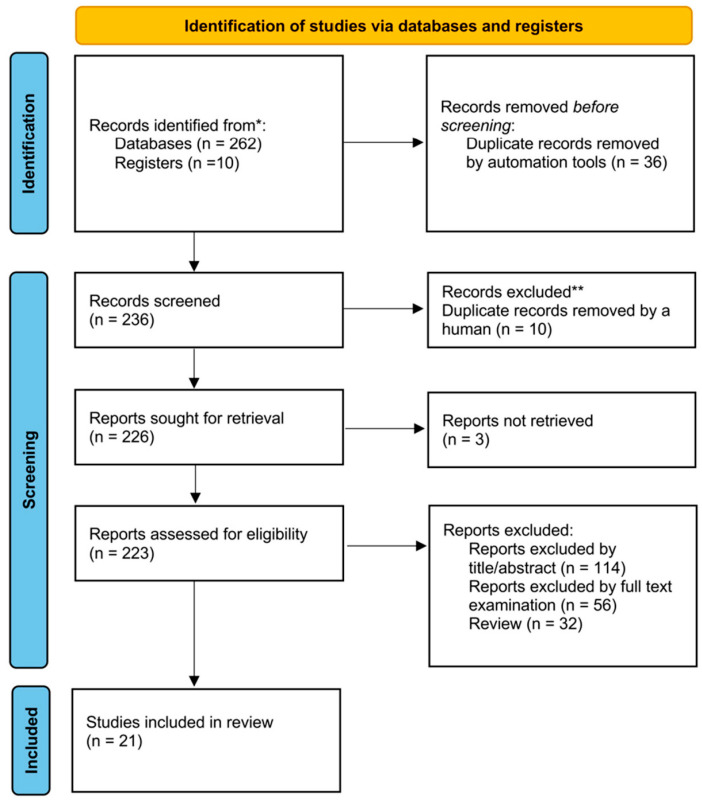
PRISMA 2020 flow diagram for new systematic reviews, which included searches of databases, registers, and other sources. * Records were identified from electronic databases and registers, including PubMed, Embase, Web of Science, CENTRAL, and ClinicalTrials.gov. ** Records excluded at this stage were manually identified duplicate records removed after automated deduplication.

The study selection process is illustrated in the PRISMA flow chart (Figure 1). A total of 272 records were initially identified, including 262 from databases and 10 from registers. After the removal of 36 duplicate records using automation tools (RefWorks (Clarivate.com, cloud-based reference management service; accessed on 19 November 2025)), 236 records were screened. During the screening process, 10 additional duplicate records were identified and removed manually. The remaining 226 reports were sought for retrieval, and 3 reports could not be retrieved. Therefore, 223 reports were assessed for eligibility. Overall, 202 reports were excluded during the selection process, including 114 excluded on the basis of title/abstract screening, 56 excluded after full-text examination, and 32 review articles. Disagreements between reviewers were resolved by a third author (M.A.) acting as an adjudicator. Ultimately, 21 studies were included in the final review. Data were extracted independently by two reviewers (F.P. and M.A.R.) using a standardized data extraction form, and disagreements were resolved by discussion with a third reviewer (M.A.). The following data items were collected, when available: study design, country, sample size, participant characteristics, dermatologic condition, intervention and comparator details, iontophoresis parameters, follow-up duration, efficacy outcomes, pharmacologic performance outcomes, and adverse events. Additional variables relevant to the descriptive subgroup synthesis were also extracted when available, including drug physicochemical characteristics, reported or inferred ionic status, molecular weight class, polarity, electrode area, current density, treatment duration, treatment frequency, and cumulative exposure. When these variables were not reported or could not be reliably inferred, they were recorded as not reported. When studies reported multiple outcome measures or time points, the clinically relevant disease-specific efficacy outcomes and safety data reported by the original authors were summarized. Missing or unclear details were recorded as not reported. No imputation of missing data was performed; data were extracted and reported as presented in the original publications. Formal inter-rater agreement statistics were not prospectively calculated; however, duplicate independent review and third-reviewer adjudication were used throughout to minimize selection and extraction errors. A list of reports assessed in full text and excluded, together with the main reason for exclusion, is provided in Supplementary Table S1.

### 2.5. Quality Assessment

**Figure 2:**
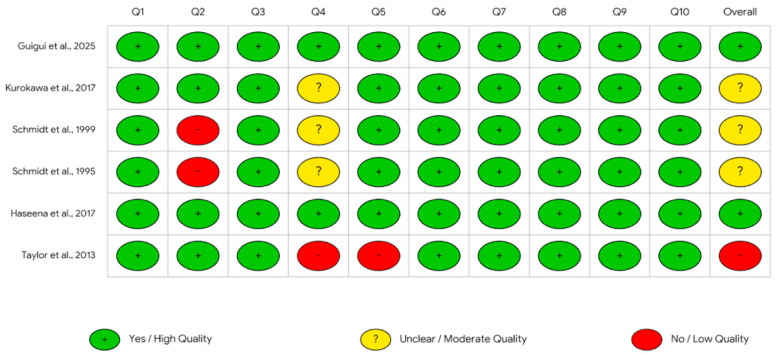
Quality assessment of the included non-randomized studies using the Joanna Briggs Institute (JBI) checklist [7,8,9,10,11,12]. Green circles with “+” indicate yes/high quality; yellow circles with “?” indicate unclear/moderate quality; red circles with “−” indicate no/low quality.

**Figure 3:**
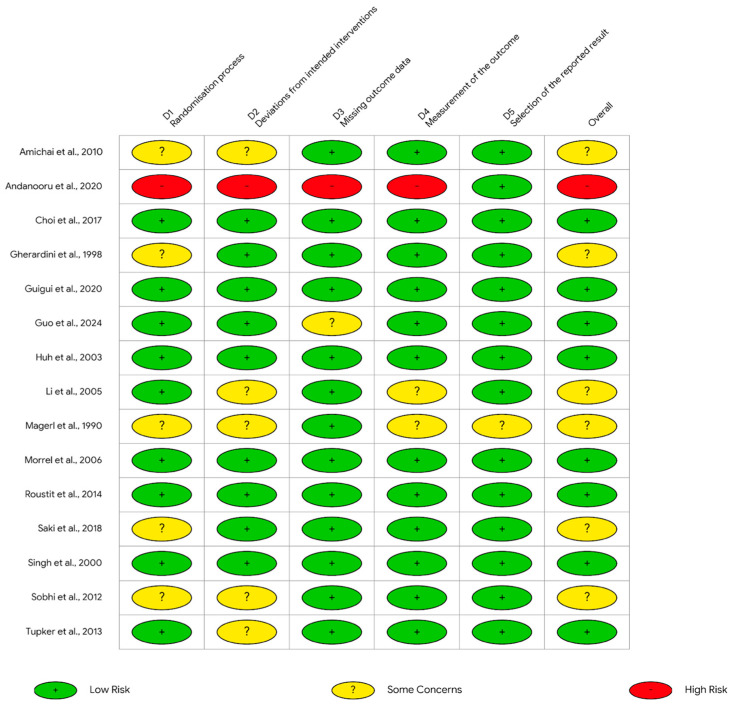
Risk-of-bias assessment of the included randomized studies using version 2 of the Cochrane Risk of Bias tool (RoB 2) [13,14,15,16,17,18,19,20,21,22,23,24,25,26,27]. Green circles with “+” indicate low risk; yellow circles with “?” indicate some concerns; red circles with “−” indicate high risk.

The methodological quality of the selected studies was independently assessed by two reviewers (F.P. and M.A.R.). Compared with the original protocol, the planned quality assessment approach was amended by replacing QUADAS-2 with study-design-specific tools, namely RoB 2 for randomized trials and the JBI checklist for non-randomized studies, because QUADAS-2 is intended for diagnostic accuracy studies. Accordingly, version 2 of the Cochrane risk-of-bias tool (RoB 2) was used for randomized clinical trials (RCTs) (15 in total), and the Joanna Briggs Institute (JBI) checklist was used for the other studies (6 in total), as shown in Figure 2 and Figure 3. Any discrepancies in the initial assessment were primarily resolved through discussion to reach a consensus; in cases where agreement could not be reached, a third reviewer (M.A.) was consulted for final adjudication. Formal inter-rater agreement statistics for risk-of-bias assessment were not calculated. The results of the methodological quality assessment are summarized in Figure 2 for non-randomized studies (JBI) and in Figure 3 for randomized trials (RoB 2).

### 2.6. Data Synthesis and Analysis

A narrative synthesis was conducted. Studies were grouped for synthesis according to dermatologic condition, drug delivered, comparator, and iontophoresis protocol. To better characterize sources of heterogeneity, studies were further summarized according to dermatologic disease category, drug physicochemical characteristics, and iontophoresis protocol parameters. Drug-related variables included ionic status and molecular weight class when reported or inferable, whereas protocol-related variables included current density, treatment duration, polarity, and treatment frequency. Given the heterogeneity of outcomes, comparators, and follow-up durations, these subgroup comparisons were descriptive rather than quantitative. Effect measures were reported as presented in the original studies, including mean or median changes for continuous outcomes and proportions for categorical outcomes, with *p* values and confidence intervals reported when available. Results of individual studies were tabulated in Table 1 and Table 2 and described narratively in the text. Because of substantial heterogeneity in populations, interventions, comparators, follow-up durations, and outcome definitions, no meta-analysis, quantitative subgroup analysis, sensitivity analysis, or quantitative exploration of heterogeneity was performed. Accordingly, pooled heterogeneity statistics such as Chi^2^ and I^2^ were not estimated, because the included studies were not considered sufficiently comparable for quantitative synthesis, including within condition-specific subgroups. A formal assessment of reporting bias was not performed because the number of studies per condition was limited and no quantitative synthesis was undertaken. A formal assessment of certainty of evidence (e.g., GRADE) was not performed because outcomes were reported inconsistently across heterogeneous study designs, interventions, comparators, and effect measures, which limited the feasibility and interpretability of outcome-level certainty ratings.

## 3. Results

### 3.1. Study Selection

A total of 272 records were identified through a systematic search. After automated duplicate removal (*n* = 36), 236 records were screened. A further 10 duplicate records were removed manually, leaving 226 reports sought for retrieval; 3 reports were not retrieved, and 223 reports were assessed for eligibility. Twenty-one studies met the eligibility criteria and were included in this review [7,8,9,10,11,12,13,14,15,16,17,18,19,20,21,22,23,24,25,26,27]. The study selection process is shown in Figure 1. Detailed reasons for exclusion of full-text reports that appeared potentially eligible are reported in Supplementary Table S1.

### 3.2. General Characteristics of the Included Studies

Studies were published from 1990 to 2025. Studies were conducted across Europe (Italy, France, Germany, Austria, Sweden, the Netherlands) [8,10,11,17,18,19,23,24,25], Asia (South Korea, Japan, India, China) [7,12,13,14,16,20,21,22,27], and North America [9,15,26]. The included studies comprised 15 RCTs [13,14,15,16,17,18,19,20,21,22,23,24,25,26,27] and 6 non-RCTs [7,8,9,10,11,12]. Sample sizes ranged from small proof-of-concept studies [8] (<10 participants) to randomized trials enrolling >100 participants [15]. The included studies investigated the use of iontophoresis across a wide range of dermatologic conditions, including inflammatory dermatoses (psoriasis, eczema) [7,13,14,23], pigmentary disorders (melasma, post-inflammatory hyperpigmentation) [9,20,21,22], infectious diseases (herpes labialis, onychomycosis) [15,27], ulcerative/vascular disorders (venous ulcers, diabetic foot ulcers, systemic sclerosis-related digital ulcers) [8,17,18,19], acne and scarring [10,11,12], and actinic keratosis [16]. Three studies investigated the safety and physiological properties of iontophoresis [24,25,26]. Follow-up durations varied widely depending on study design, ranging from minutes to hours in acute physiological or pharmacokinetic studies [8,18,24,25,26] to weeks or months in clinical efficacy trials [7,9,10,12,13,14,15,16,17,19,20,21,22,23,27], and up to years in long-term cosmetic studies [11]. Across all studies, iontophoresis was generally well tolerated, with the most frequently reported adverse effects being mild and transient, including localized erythema, tingling, burning sensations, and temporary discomfort at the application site. No serious adverse events were reported. Rare instances of superficial skin burns [18] or increased irritation were observed in studies with higher current densities [18] or surfactant pre-treatment [25]. The main characteristics and outcomes of the included clinical studies are summarized in Table 1.

**Table 1:** Clinical studies on iontophoresis-assisted drug delivery in dermatology, grouped by disease category.

Study	Participants	Condition	Intervention	Comparator	Protocol	Current Density	Polarity	Drug Charge	Main Results	Follow-Up	Adverse Events
Inflammatory dermatoses											
Saki et al. [13], 2018	*n* = 16; mean age 34.3 y; F/M = 11/5	Nail psoriasis	Triamcinolone acetonide iontophoresis	Topical calcipotriol/betamethasone dipropionate	20 min/session; monthly for 6 months; pulsed delivery with 0.2-s pulse duration	NR; 4 mA total current, pulsed, 20 min	NR	Neutral/non-ionized, I; triamcinolone acetonide	Both groups improved significantly; no significant between-group difference in nail bed, nail matrix, or total NAPSI	6 months	NR
Haseena et al. [7], 2017	*n* = 28; mean age 42.2 y; F/M = 11/17	Palmoplantar psoriasis	Methotrexate iontophoresis	Coal tar ointment	15 min/session; 6 sessions	NR; 5–10 mA total current, 15 min	Cathodal, R	Negative/anionic, R; methotrexate	Greater ESIF improvement with methotrexate iontophoresis than coal tar (63.9% vs. 47.7%; *p* < 0.001)	8 weeks	Mild pruritus in 1 patient; no hematologic, renal, or hepatic abnormalities
Andanooru et al. [14], 2020	*n* = 50; mean age 42.3 y; F/M = 22/28	Palmar psoriasis	Methotrexate iontophoresis	Clobetasol propionate 0.05% ointment	Once weekly for 6 weeks	0.004–0.217 mA/cm^2^, mean ≈ 0.081 mA/cm^2^, C from 0.2–12 mA and 8.5 × 6.5 cm electrode	Cathodal, R	Negative/anionic, R; methotrexate	Satisfactory improvement in 32% vs. 48%; no significant between-group difference (*p* = 0.25)	6 weeks	Burn injury in 48% of iontophoresis group; none in comparator group
Tupker et al. [23], 2013	*n* = 48 in 3 groups	Chronic foot eczema	Iontophoresis + bath-PUVA	Bath-PUVA alone; topical fluticasone	10 min/session; 3 times/week for 8 weeks	NR; max 30 mA total, 10 min	NA/NR	NA; tap-water iontophoresis, no iontophoretic drug	Eczema score and DLQI improved in all groups; no significant between-group difference	8 weeks	Burning and mild erythema in PUVA groups; none in steroid group
Infectious diseases											
Morrel et al. [15], 2006	*n* = 200; mean age 34 y; F/M = 114/86	Herpes labialis	Acyclovir 5% cream iontophoresis	Placebo cream + iontophoresis	Single 10-min application of 250 mg acyclovir 5% cream; clinic-initiated at erythema or papule/edema stage	NR; 10 min treatment, current not reported	Anodal/positive active electrode, R	NR; acyclovir charge not specified	Median healing time shorter with active treatment (113 h vs. 148 h; *p* = 0.02); greater benefit at erythema stage (49 h vs. 120 h; *p* < 0.03)	10 days	Electrical sensation, skin burn, transient erythema; all infrequent
Amichai et al. [27], 2010	*n* = 38; mean age 46.2 y; F/M = 16/22	Onychomycosis	Terbinafine 1% gel patch with iontophoresis	Identical patch without current	Patch applied overnight for 6–8 h/day, 5 days/week for 4 weeks	0.1 mA/cm^2^, R; 100 µA/cm^2^	Anodal/positive active electrode, R	Positive/cationic, I; terbinafine HCl at gel pH 4.6	Higher nail terbinafine concentrations, better mycologic outcome, and greater clinical improvement with active iontophoresis	8 weeks	Mild tingling; mild local irritation in 2 patients; no systemic AEs
Ulcerative and vascular disorders											
Guigui et al. [8], 2025	*n* = 4; mean age 66 y; F/M = 1/3	Chronic diabetic foot ulcers	Treprostinil hydrogel iontophoresis	None	Single 30-min ascending-dose session	0.2 mA/cm^2^, R; 30 min	Cathodal, R	Negative/anionic, I; treprostinil	Technically feasible up to 0.25 mg/mL; plasma levels below lower limit of quantification at all time points	Up to 8 h, plus 48-h safety check	Four AEs total; judged unrelated; no iontophoresis-related safety concerns
Gherardini et al. [17], 1998	*n* = 66; mean age 60.8 y; F/M = 41/24	Venous ulcers	CGRP/VIP iontophoresis	Placebo iontophoresis	20 min/session; 3 sessions/week for 12 weeks	NR	NR	NR; CGRP/VIP peptide charge not stated	Greater ulcer area reduction (74% vs. 44%) and higher complete healing (60% vs. 41%); *p* < 0.05	12 weeks	NR
Guigui et al. [18], 2020	Healthy volunteers: *n* = 12; SSc patients: *n* = 5	Systemic sclerosis-related digital ulcers	Treprostinil hydrogel iontophoresis	Placebo hydrogel iontophoresis	Healthy volunteers: 20–120 min/session; SSc-DU patients: 60 min/session	NR as mA/cm^2^; individualized current/charge protocol	Cathodal, R	Negative/anionic, I; treprostinil	Increased cutaneous blood flow in healthy volunteers; acceptable tolerability in SSc-DU patients; no healing outcomes assessed	Up to 8 h, plus 48-h phone follow-up	Healthy volunteers: multiple grade 1–2 local AEs; SSc-DU patients: 2 mild local AEs; no systemic AEs
Roustit et al. [19], 2014	Healthy subjects: *n* = 22; SSc patients: *n* = 12	Systemic sclerosis	Treprostinil iontophoresis	Placebo iontophoresis	Sequential dose-escalation protocols; finger-pad application in SSc patients	Reported as 40–240 mC/cm^2^; 240 mC/cm^2^ over 20 min ≈ 0.20 mA/cm^2^, C	Cathodal, R	Negative/anionic, I; treprostinil	Significant increase in skin blood flow in healthy subjects (*p* = 0.006) and SSc patients (*p* = 0.023); lower response in severe microangiopathy	10 days	Transient erythema; occasional petechiae/hematoma; mild headache in 2 patients
Pigmentary disorders											
Huh et al. [20], 2003	*n* = 29; mean age 36.5 y; all female	Melasma	Vitamin C iontophoresis	Placebo iontophoresis	8 min/session; weekly for 12 weeks	NR; 0.3–0.5 mA total, 8 min	Cathodal/negative active electrode, R	Negative/anionic, R; vitamin C derivative/MAP	Significant improvement on treated side by objective colorimetry; significant between-side difference (*p* = 0.03)	12 weeks	Mild and transient electrical shock, itching, erythema, burning, and dryness
Taylor et al. [9], 2013	*n* = 35; mean age 47.6 y; F/M = 34/1	Melasma/post-inflammatory hyperpigmentation	Full-face iontophoresis mask + vitamin C derivative + skin-care regimen	Baseline comparison	1 h/session; 3 times/week for 1–2 months	0.0018 mA/cm^2^, R; mask output 1.8 µA/cm^2^	Cathodal/negative active pad, R	Negative/anionic, R; vitamin C	Mean pigmentation improvement 73%; MASI mean improvement 15.7; texture +62%; wrinkles +39%	Mean 26 months	Minor acne breakout in 1 patient; treatment generally well tolerated
Sobhi et al. [21], 2012	*n* = 14; mean age 37.5 y; all female	Melasma	Nanosome vitamin C iontophoresis	Glycolic acid 70% peel	0.5 mL/session; 10 min/session; 6 sessions	NR; reported as 5 mA × 10 min, area NR	Cathodal/negative mode, R	Negative/anionic, I; vitamin C formulation	Greater MASI reduction on vitamin C side than peel side, but no significant between-side difference due to small sample	6 weeks	Mild erythema and tingling
Guo et al. [22], 2024	*n* = 30; mean age 39 y; all female	Melasma	Tranexamic acid essence + iontophoresis	Placebo iontophoresis	Twice weekly for 3 months	NR; voltage/power reported, no current density	NR	Zwitterionic/pH-dependent, I; tranexamic acid, not specified in paper	Significant MASI reduction and increased skin brightness versus placebo (*p* < 0.05; L-value *p* = 0.037)	12 weeks	None reported
Acne-related conditions and scarring											
Schmidt et al. [10], 1995	Estriol group: *n* = 18, all female; tretinoin group: *n* = 28, F/M = 19/9	Atrophic acne scars	Estriol iontophoresis or tretinoin iontophoresis	Baseline comparison	15 min/session; twice weekly for 3 months	NR; 3 mA total, 15 min	Cathodal for acidic solutions, R	Estriol: neutral/weakly polar, I; tretinoin: negative/anionic, I	Improvement in 100% of estriol group and 93% of tretinoin group; no systemic hormonal changes	3 months	Estriol: none; tretinoin: dryness and dermatitis
Schmidt et al. [11], 1999	*n* = 32; mean age 31.5 y; F/M = 19/13	Atrophic acne scars	Tretinoin iontophoresis	Baseline comparison	20 min/session; twice weekly for 3 months	NR; 3 mA total, 20 min	Cathodal, R	Negative/anionic, I; tretinoin/retinoic acid	Significant decrease in scar depth in 94%; no significant increase in epidermal thickness or proliferation markers	6–12 months	Transient flushing; dryness in 32%; fine scaling in 4 cases
Kurokawa et al. [12], 2017	*n* = 31; mean age 22.6 y; F/M = 22/9	Post-inflammatory hyperpigmentation, erosions/red papules, and atrophic acne scars in acne vulgaris	Chemical peeling followed by iontophoresis	Baseline comparison	3–4 cycles at 1–2-month intervals; total treatment duration 3–6 months	NR; current not reported	NR	Negative/anionic phosphate derivatives, I; APPS/TPNa	Excellent improvement in most PIH and erosion/red papule cases; less effect on atrophic scars	3–6 months	Mild redness and irritation in 4 cases
Premalignant lesions											
Choi et al. [16], 2017	*n* = 41; mean age 69 y; F/M = 22/19	Actinic keratosis	Iontophoresis-assisted AFL-PDT with short incubation	Conventional AFL-PDT (2-h or 3-h incubation)	10 min iontophoresis followed by further incubation; MAL after Er:YAG AFL and red-light illumination	0.50 mA/cm^2^, R; 10 min	Anodal/positive active electrode, R	Positive/cationic, R; MAL	Complete response comparable to conventional 3-h protocol at 3 and 12 months; lower recurrence than 2-h conventional protocol	3–12 months	Mild-to-moderate local phototoxic reactions; no systemic AEs

Abbreviations: AE, adverse event; AFL-PDT, ablative fractional laser photodynamic therapy; C, calculated; CGRP, calcitonin gene-related peptide; DLQI, Dermatology Life Quality Index; ESIF, erythema-scaling-induration-fissuring; I, inferred; MAL, methyl aminolevulinate; MASI, Melasma Area and Severity Index; NA, not applicable; NAPSI, Nail Psoriasis Severity Index; NR, not reported; PIH, post-inflammatory hyperpigmentation; PUVA, psoralen plus ultraviolet A; R, reported; SSc, systemic sclerosis; SSc-DU, systemic sclerosis-related digital ulcer; VIP, vasoactive intestinal polypeptide.

### 3.3. Methodological Quality and Risk of Bias

Among the 15 randomized studies assessed using RoB 2, 57 of 75 domain-level judgments were rated as low risk, 14 as some concerns, and 4 as high risk, corresponding to 76.0%, 18.7%, and 5.3%, respectively. At the study level, 8/15 randomized studies were judged overall low risk, 6/15 had some concerns, and 1/15 was judged high risk. High-risk judgments were concentrated in one trial, whereas some concerns most commonly involved the randomization process and deviations from intended interventions.

Among the 6 non-randomized studies assessed using the JBI checklist, 53 of 60 item-level judgments were rated as yes/high quality, 3 as unclear/moderate quality, and 4 as no/low quality, corresponding to 88.3%, 5.0%, and 6.7%, respectively. Overall JBI ratings were high quality in 2/6 studies, moderate/unclear in 3/6, and low quality in 1/6.

A cross-cutting limitation was the small to moderate sample size of most included studies. Sample sizes ranged from small proof-of-concept studies with fewer than 10 participants to one trial enrolling 200 participants, with most studies enrolling fewer than 50 participants. This limited statistical precision and reduced the robustness of condition-specific conclusions, particularly within individual dermatologic indications.

### 3.4. Characteristics of the Intervention

Across the included studies, iontophoresis was used as a local technique for transdermal drug delivery, either as a sole intervention or in comparison with passive topical application and standard therapies. In some studies, iontophoresis was used as an adjuvant technique combined with other treatment methods, such as ablative fractional laser photodynamic therapy [16], chemical peeling [12], and bath-psoralen plus ultraviolet A (PUVA) [23].

Several pharmacologic agents were delivered through iontophoresis, including anti-inflammatory and immunomodulatory drugs (triamcinolone acetonide [13], methotrexate [7,14]), antiviral agents (acyclovir) [15], antifungal agents (terbinafine) [27], depigmenting agents (vitamin C [9,20,21], tranexamic acid [22]), vasoactive compounds (treprostinil [8,18,19], calcitonin gene-related peptide, vasoactive intestinal peptide [17]), and retinoids or hormonal agents (tretinoin [10,11], estriol [10]). The formulation of these agents varied widely and included solutions, gels (acyclovir [15] and treprostinil [8,18,19]), creams (topical corticosteroids [13], tretinoin [10,11] and estriol [10]), hydrogels, and advanced delivery systems (nanosome-based vitamin C formulations [21] and full-face iontophoresis masks [22]).

The parameters of iontophoresis varied across studies. The current densities ranged from 0.1 to 0.5 mA/cm^2^, which were considered low to moderate and were chosen based on the physical and chemical characteristics of the drug being delivered and the anatomical site treated.

The duration of application ranged from 10 to 30 min in most studies to several hours with longer exposure times or single-ascending dose protocols in safety studies [8,18,19], and experimental histamine iontophoresis studies evaluating physiological responses of the skin [24]. The regimen of the treatment spanned from single sessions in safety and mechanistic studies [8,19,24] to repeated applications over weeks or months in clinical efficacy studies [7,10,11,12,13,14,20,22,27].

Comparators varied based on the clinical indication. Passive topical application was used as the primary comparator in many RCTs [13,14,15,20,22,27]. In the treatment of inflammatory dermatoses, standard therapies such as coal tar ointment [7] and bath-PUVA therapy [23] were used as active comparators. In pigmentary and cosmetic indications, iontophoresis was compared with or added to chemical peeling alone [12,21] or included in combination regimens [9]. In actinic keratosis, iontophoresis was implemented to improve the delivery of photosensitizers within protocols of photodynamic therapy, allowing for decreased incubation times compared with conventional photodynamic therapy (PDT). Several studies investigated the skin tolerability and the physiological effects of iontophoresis instead of therapeutic efficacy. These studies assessed the histamine-induced wheal and flare responses [24]_,_ the cutaneous side effects with and without surfactant pretreatments [25], and the effects of saline iontophoresis on skin barrier function and irritation across different ethnic groups [26]. The interventions were performed in outpatient settings and implemented on localized skin areas, apart from full-face applications in selected disorders of pigmentation [9].

#### Descriptive Subgroup Synthesis

When studies were examined by disease category, the most consistent signals of benefit were observed in pigmentary disorders and infectious diseases [9,12,15,20,21,22,27], whereas findings in inflammatory dermatoses were more heterogeneous [7,13,14,23]. Ulcerative and microvascular disorders showed encouraging physiologic or clinical signals, particularly when iontophoresis was used to deliver vasoactive compounds [8,17,18,19].

Regarding drug physicochemical characteristics, favorable outcomes were most frequently reported with charged or polar compounds, including vitamin C derivatives [9,12,20,21], terbinafine [27], treprostinil [8,18,19], methyl aminolevulinate [16], and tranexamic acid [22]. However, a clear relationship between ionic status, molecular weight class, and clinical response could not be established, because formulation pH, drug ionization state, electrode area, and current density were inconsistently reported across studies [7,8,9,10,11,12,13,14,15,16,17,18,19,20,21,22,23,24,25,26,27].

Protocol parameters also varied substantially. Short-to-intermediate applications of approximately 8–30 min were most common in clinical studies [7,8,10,11,13,15,16,20,21], whereas prolonged applications were mainly used in patch-based or safety studies [9,18,25,26,27]. No consistent dose–response relationship between current density, treatment duration, and efficacy could be inferred from the available data [7,8,9,10,11,12,13,14,15,16,17,18,19,20,21,22,23,24,25,26,27].

### 3.5. Description of Each Study

Because detailed protocols and outcomes are reported in Table 1 and Table 2, this section provides a concise descriptive summary of the main efficacy and safety findings by study type and indication.

#### 3.5.1. Randomized Clinical Trials

For the treatment of inflammatory dermatoses, Saki et al. [13] conducted a bilateral controlled randomized trial involving 16 patients with nail psoriasis, comparing triamcinolone acetonide delivered by iontophoresis with topical calcipotriol/betamethasone dipropionate. Both treatments produced significant reductions in the nail bed and total Nail Psoriasis Severity Index (NAPSI) scores; however, no statistically significant difference was observed between the treatment modalities. Iontophoresis was associated with earlier clinical improvement, with a reduction in NAPSI noted after the third to fourth month. In palmoplantar and palmar psoriasis, Andanooru et al. [14] randomized 50 patients to receive either methotrexate iontophoresis or clobetasol propionate 0.05% ointment. After 6 weeks, satisfactory clinical improvement (>50% reduction in the modified Palmoplantar Psoriasis Area and Severity Index) was observed in 32% of the iontophoresis group and 48% of the topical steroid group, without a statistically significant difference. Burn injuries were reported in 48% of patients in the iontophoresis group, while no adverse effects were observed in the comparator group. Tupker et al. [23] assessed the use of iontophoresis combined with bath-PUVA therapy versus PUVA alone or topical steroids for chronic foot eczema. The eczema severity score decreased significantly across all groups (10.69 → 6.87, *p* < 0.001), but no significant difference between groups was noted (*p* = 0.053). The same was true for the DLQI (Dermatology Life Quality Index) and the patients’ global impression, which improved significantly in all groups but showed no difference between groups.

For the treatment of infectious diseases, Morrel et al. [15] performed a randomized, double-blind, placebo-controlled trial including 200 patients with recurrent herpes labialis. Acyclovir delivered by iontophoresis reduced the median lesion healing time by approximately 1.5 days compared with placebo, with a greater effect observed in lesions treated at the erythema stage. For onychomycosis, Amichai et al. [27] showed that iontophoretic delivery of terbinafine resulted in significantly higher nail drug concentrations (5.69 μg/cm^2^ in active treatment vs. 1.34 μg/cm^2^ in passive treatment), improved mycological outcomes (16% positive potassium hydroxide in active treatment vs. 53% in passive treatment) and better clinical improvement (42% of the active group achieved ≥1.5 mm of new nail growth vs. 19% in passive treatment) compared with passive topical application.

For the treatment of actinic keratosis, Choi et al. [16] evaluated iontophoresis-assisted ablative fractional laser photodynamic therapy (AFL-PDT) in a randomized comparative trial. Iontophoresis-assisted short incubation AFL-PDT achieved a complete response rate comparable to that of conventional long-incubation (3-h) protocols at both 3 and 12 months (83% with iontophoresis vs. 84.3% in long-incubation conventional AFL-PDT), with lower recurrence rates (6.4% with iontophoresis vs. 8.5% with conventional AFL-PDT).

For the treatment of ulcerative and vascular conditions, Gherardini et al. [17] showed significantly greater ulcer area reduction and higher complete healing rates at 12 weeks in venous ulcers treated with calcitonin gene-related peptide (CGRP) and vasoactive intestinal polypeptide (VIP) compared with placebo iontophoresis (74% mean area reduction in the CGRP/VIP group vs. 44% mean area reduction in placebo iontophoresis) with a *p* < 0.05. Regarding the healing rates, 60% of subjects in the CGRP/VIP group vs. 41% in the control group achieved complete healing at 12 weeks.

Guigui et al. (2020) [18] conducted a randomized safety study of treprostinil hydrogel iontophoresis for systemic sclerosis-related digital ulcers. Twelve healthy volunteers and 5 patients with systemic sclerosis were assessed. In healthy volunteers, treprostinil significantly increased cutaneous blood flow at the leg and sole sites, with a trend observed on the fingers. In patients with systemic sclerosis-related digital ulcers (SSc-DU), iontophoresis was acceptably tolerated, with only 2 mild local adverse effects that both resolved, but no healing outcomes were measured. In healthy volunteers, 60 local adverse effects, all adverse events (AE) graded 1–2 (mild to moderate), were observed: burns, pain, erythema, and pruritus. Roustit et al. [19] reported significant increases in skin blood flow in healthy subjects (*p* = 0.006) and in systemic sclerosis patients (*p* = 0.023) after the delivery of treprostinil trough iontophoresis. Reduced responsiveness was noted in patients with a late capillaroscopy pattern (severe microangiopathy).

For the treatment of melasma, multiple randomized trials evaluated the efficacy of depigmenting therapies coupled with iontophoresis. Huh et al. [20] performed a placebo-controlled trial of vitamin C iontophoresis for the treatment of melasma. The side treated with vitamin C iontophoresis showed a significant decrease in the objective colorimetry luminance value (from 4.60 to 2.78 at 12 weeks, *p* = 0.002), while no significant changes were noted on the placebo side (from 4.45 to 3.87, *p* = 0.142), with a significant between-group difference (*p* = 0.03). Sobhi et al. [21] compared glycolic acid 70% peeling versus nanosome vitamin C iontophoresis in the treatment of melasma. Greater Melasma Area and Severity Index (MASI) reduction was observed with nanosome vitamin C iontophoresis (mean decrease 3.535, *p* < 0.0001) compared to glycolic acid peeling (mean decrease 1.757, *p* = 0.001); however, due to the small sample size, the between-group difference did not reach significance. Guo et al. [22] demonstrated that tranexamic acid essence combined with iontophoresis produced significant reductions in MASI scores (−0.10 ± 0.12) compared with placebo (−0.02 ± 0.09), with a *p* < 0.05. Improvements in skin brightness were also significant, with an L-value increase from 61.32 to 63.32 in the tranexamic acid group vs. a decrease from 63.50 to 62.44 in the placebo group, with a *p* = 0.037. All treatments were well tolerated, with only mild and transient local adverse effects.

The physiologic and safety-focused studies are summarized in Table 2. Physiological and safety-focused randomized studies assessed the cutaneous responses to iontophoresis. Magerl et al. [24] characterized histamine-induced wheal and flare responses delivered via iontophoresis, demonstrating reproducibility with variability related to season (larger responses in warmer months), gender (higher flare magnitude in men), and anatomical site (flare amplitude larger in the forearm compared with the thigh). Li et al. [25] compared the cutaneous side effects of iontophoresis with and without pretreatment with surfactant. The findings showed that iontophoresis alone caused minimal irritation, while surfactant pretreatment (0.5% sodium dodecyl sulfate) significantly increased erythema and transepidermal water loss, indicating worsened irritation. Barrier function returned to normal within 24 h. Finally, Singh et al. [26] investigated the effect of saline iontophoresis on skin barrier function and irritation in four ethnic groups and showed that transepidermal water loss increased slightly only at 60 min after iontophoresis, returning to baseline by 120 min (highest in Caucasians, lowest in Blacks). Regarding cutaneous irritation, erythema was observed at active electrodes in all ethnicities immediately after application, resolving completely by 24 h (with irritation scores lower in Black participants).

**Table 2:** Physiologic and safety-focused studies on iontophoresis in dermatology.

Study	Participants	Condition	Intervention	Comparator	Protocol	Current Density	Polarity	Drug Charge	Main Results	Follow-Up	Adverse Events
Magerl et al. [24], 1990	Winter experiment: *n* = 48; summer: *n* = 23; body regions: *n* = 20; itch discrimination: *n* = 6	Cutaneous physiologic response	Histamine iontophoresis	Within-subject comparison	Acute testing at multiple body sites; seasonal/itch subexperiments; 5-mm chamber	NR; reported charge range 0.05–30 mC; test pulse 10 mC; 5 mm chamber	Anodal histamine delivery	Cationic	Responses varied by season, gender, and body region; stronger responses in warmer months and at selected sites	Acute assessment	Minimal transient erythema and mild burning; no systemic AEs
Li et al. [25], 2005	*n* = 24 healthy volunteers; mean age 31 y; F/M = 12/12	Cutaneous side effects	Iontophoresis with or without SDS pretreatment	Passive occlusion; iontophoresis alone; surfactant alone	Single 3 h exposure; SDS 0.5% pretreatment for 1 h where applicable	0.25 mA/cm^2^; 3 h; 2.5 cm^2^ cell; total current 0.625 mA	Anode/cathode system; surfactant pretreatment at anodal site	NA; no active drug	Iontophoresis alone caused minimal irritation; SDS pretreatment increased erythema and TEWL; barrier recovery within 24 h	8 days	Mild erythema with iontophoresis alone; more marked irritation with SDS pretreatment
Singh et al. [26], 2000	*n* = 40 healthy volunteers; 10 each Caucasian, Black, Hispanic, Asian; equal sex distribution	Cutaneous irritation/barrier function	Saline iontophoresis	Saline patch alone	Single 4 h patch exposure; post-treatment irritation/barrier assessment	0.20 mA/cm^2^; 4 h; 6.5 cm^2^ patch; total current 1.3 mA	Central anode/perimeter cathode	NA; saline only	TEWL increased slightly at 60 min then returned to baseline; erythema resolved by 24 h; irritation somewhat lower in Black participants	24 h	Mild transient erythema; occasional transient papules; no systemic AEs

Abbreviations: AE, adverse event; NA, not applicable; NR, not reported; SDS, sodium dodecyl sulfate; TEWL, transepidermal water loss.

#### 3.5.2. Non-Randomized Clinical Trials

In acne-related conditions, Schmidt et al. (1995) [10] compared the clinical efficacy of tretinoin iontophoresis vs. estriol iontophoresis in the treatment of atrophic acne scars and observed improvement in 93% of patients in the tretinoin group and in 100% of patients in the estriol group, with no hormonal changes noticed in the latter group. In the same field, Schmidt et al. (1991) [11] demonstrated a significant decrease in scar depth in 94% of patients treated with tretinoin iontophoresis, while epidermal thickness and proliferation markers were not significantly increased at the end of treatment. Kurokawa et al. [12] evaluated the efficacy of chemical peeling with 20% glycolic acid followed by iontophoresis for the treatment of post-inflammatory hyperpigmentation (PIH), erosions with red papules and atrophic acne scars in acne vulgaris based on a 6-point scale rating, with 4 indicating 75% to 100% improvement and −1 indicating worsening. The study demonstrated an excellent improvement of PIH in 26 of 31 cases (mean score 3.7), of erosions with red papules in 15 of 21 cases (mean score 3.7), while in atrophic acne scars the treatment was less effective, with 5 of 20 cases showing no change (mean score 2.3). Only mild, transient cutaneous side effects such as dryness and erythema were reported.

In palmoplantar psoriasis, Haseena et al. [7] conducted a pilot study comparing methotrexate iontophoresis with coal tar ointment, demonstrating a 63.95% mean improvement in the erythema, scaling, induration, and fissuring (ESIF) score in the methotrexate iontophoresis group compared with 47.66% in the coal tar ointment group. The between-group difference in improvement was statistically significant (*p* < 0.001). No systemic toxicity was noted.

In the treatment of melasma and post-inflammatory hyperpigmentation, Taylor et al. [9] investigated the efficacy of applying vitamin C with a full-face iontophoresis mask plus a mandelic/malic acid skin care regimen. The study reported a mean improvement from baseline of 73% using a 1–4 scale assessed by 4 independent observers, with 1 being slightly better and 4 being clear or almost clear. Mean improvements in skin texture and wrinkles were 62% and 39% respectively. The MASI assessed in 4 regions (forehead, right and left malar, and chin) showed a mean improvement of 15.7. The treatment was well tolerated.

Guigui et al. (2025) [8] conducted a single ascending dose safety study of treprostinil iontophoresis in diabetic foot ulcers, showing technical feasibility at up to 0.25 mg/mL. Minimal systemic absorption was demonstrated, with all plasma concentrations below the lower limit of quantification at all time points up to 8 h, and no iontophoresis-related safety concerns.

## 4. Discussion

### 4.1. Inflammatory Chronic Dermatoses

The therapeutic goal of using iontophoresis in inflammatory dermatoses is to maximize drug concentration at the pathological site while reducing potential treatment-associated toxicity and systemic absorption. An ideal prototype for this approach is represented by psoriasis, especially when the therapy needs to be applied to structurally complex body sites such as the nails and palmoplantar surfaces. Inconsistent effectiveness has been noted with the use of conventional topical drugs, probably due to the structure of the nail plate and hyperkeratotic palmar skin, which constitute a significant obstacle to passive diffusion.

In psoriasis, the available studies suggest that iontophoresis may be most useful in anatomically challenging sites, such as nails and palmoplantar skin, where passive topical penetration is limited [7,13,14]. Although clinical superiority over active topical comparators was not consistently demonstrated, iontophoresis may reduce the need for daily topical application, potentially improving adherence and reducing interference with patients’ daily routines [2]. However, its cost-effectiveness remains context-dependent, as these potential advantages must be balanced against device cost, availability, and usability. Therefore, future studies should include patient-reported adherence, treatment burden, access to equipment, and formal cost-effectiveness outcomes in addition to clinical efficacy and safety endpoints [2].

Nevertheless, in chronic foot eczema, no additional improvement was observed when combining bath-PUVA and tap-water iontophoresis [23], which signifies that the therapeutic benefit of iontophoresis greatly relies on the pathological context. In eczema, central features such as barrier malfunction and dysregulation of the inflammatory response explain why enhancing only transdermal penetration may not be enough to significantly alter disease activity. These different outcomes show that iontophoresis acts as a therapeutic enhancer whose effectiveness relies on the pharmacodynamics of the agent and the substrate’s pathophysiological state.

Overall, evidence on the use of iontophoresis in inflammatory dermatoses indicates that the greatest advantage is achieved when the principal limiting element is drug penetration and when significant toxicity is expected with the use of systemic therapy.

### 4.2. Pigmentary Disorders

Within the reviewed dermatological disorders, pigmentary disorders display the most consistent benefit with the highest reproducibility from iontophoresis-assisted therapy. Melasma is characterized by chronic hyperactivation of melanocytes and recurrence. Management of this disorder proves difficult due to the poor stability and penetration of depigmenting agents.

Across pigmentary disorders, the available studies consistently suggest that iontophoresis can enhance the delivery of depigmenting agents such as vitamin C and tranexamic acid, leading to objective improvement in melasma and post-inflammatory hyperpigmentation [9,12,20,21,22]. From a mechanistic standpoint, this approach may be particularly relevant for hydrophilic or unstable molecules with limited passive permeability, allowing improved local bioavailability while avoiding the risks associated with more invasive or systemic approaches.

A significant finding is represented by the consistent results of these studies regarding the safety profiles, which are shown to be favorable even in darker Fitzpatrick skin types, where a major concern is the appearance of post-inflammatory hyperpigmentation. This demonstrates that the use of iontophoresis presents an alternative where invasiveness and irritation are minimal in comparison to aggressive peeling or laser-focused approaches, with a diminished risk of developing rebound pigmentation. The combined data place pigmentary conditions among the most promising domains in which iontophoresis can be used to assist in the delivery of therapies.

### 4.3. Infectious Diseases

In infectious dermatological conditions, therapy often presents a challenge related to attaining an appropriate drug concentration locally due to the anatomical sites often being shielded. This is directly addressed by iontophoresis as it allows the delivery of a focused, high-concentration dose while avoiding systemic exposure.

In recurrent herpes labialis, administering a single dose of acyclovir through iontophoresis reduced healing time significantly, especially when drug delivery was done early in lesion development [15]. This emphasizes the significance of rapidly achieving an inhibitory antiviral concentration during the active phase of viral replication that is narrow. Likewise, when treating distal lateral subungual onychomycosis with iontophoretic terbinafine, higher local concentrations and better mycological outcomes were achieved compared to passive application [27].

These findings are particularly relevant for anatomically protected sites such as the nail plate, where passive topical delivery is often inadequate. In this setting, iontophoresis may help convert agents with limited local penetration into feasible topical or transungual therapies, potentially reducing reliance on systemic treatment [27].

### 4.4. Ulcers and Microvascular Disorders

In the setting of chronic ulcers and microvascular disorders, the central pathophysiology is characterized by impaired perfusion and tissue hypoxia. The studies included in this review suggest that the benefits of iontophoresis in these conditions are defined not only by improved drug delivery but also by the modulation of local vascular responses.

In chronic venous ulcers, iontophoresis of CGRP/VIP (calcitonin gene-related peptide/vasoactive intestinal peptide) significantly enhanced ulcer surface reduction compared with placebo iontophoresis [17], probably through complementary vasodilatory and angiogenic effects. In diabetic foot ulcers, iontophoresis combined with treprostinil showed feasibility and minimal systemic exposure [8], while localized delivery of treprostinil in systemic sclerosis-associated ulcers increased perfusion to the digits and avoided adverse effects associated with systemic exposure to prostacyclins [18,19].

A particularly remarkable finding across these studies is the beneficial ratio between dermal and systemic drug concentration accomplished via iontophoresis. Vasodilatory side effects often limit the administration of prostacyclin analogues. This barrier can be bypassed by the local delivery of agents through iontophoresis. However, microvascular responses are variable, especially in cases of advanced systemic sclerosis, which suggests that the response to therapy may be limited by structural vascular injury. These results underline the need for optimizing the current density and stratifying patients to achieve better outcomes in future trials.

### 4.5. Scarring and Structural Dermal Damage

Delivery of tretinoin or estriol through iontophoresis caused considerable and long-lasting clinical improvement in the treatment of atrophic acne scars [10,11]. Curiously, no quantifiable increase in collagen deposition was consistently noted, demonstrating that clinical efficacy may be due to subtle remodeling in the dermal layer that staining techniques cannot fully capture.

The persistence of scar flattening months after the discontinuation of treatment supports true structural remodeling and argues against the transient appearance of edema. These findings are likely determined by the ability of iontophoresis to enhance the drug concentration in the dermis above that attainable with passive topical application, potentially generating an effect similar to that of a localized drug depot. The benefits this modality offers compared with invasive resurfacing methods are associated with it being non-ablative and requiring a short downtime. However, to validate the mechanism of long-term skin reorganization, controlled trials need to be completed.

### 4.6. Photodynamic Therapy and Oncologic Dermatology

In PDT, phototoxic effectiveness is directly determined by the concentration and distribution of the photosensitizing agent. To modulate these parameters, iontophoresis has been considered a tool that allows precise delivery. For the treatment of actinic keratosis, iontophoresis in conjunction with shortened-incubation AFL-PDT accomplished clinical efficacy that can be compared to conventional longer protocols [16], suggesting more efficient resource utilization and enhanced performance without compromising results.

### 4.7. Safety and Cutaneous Physiology

Across the included studies, iontophoresis was generally associated with mild and transient local adverse events, most commonly erythema, tingling, burning sensation, irritation, dryness, or temporary discomfort at the application site [9,10,11,15,18,20,21,25,26,27]. No serious treatment-related systemic adverse events were reported across the included clinical studies [7,8,9,10,11,12,13,14,15,16,17,18,19,20,21,22,23,24,25,26,27]. However, tolerability was not uniform across protocols. The most notable safety signal was reported by Andanooru et al., who found that burn injuries occurred in 48% of patients receiving methotrexate iontophoresis for palmar psoriasis, whereas no adverse effects were observed in the topical clobetasol comparator group [14]. In contrast, studies using lower-intensity or more controlled protocols generally reported only mild local reactions [9,20,21,25,26,27]. Although adverse events appeared more likely with prolonged exposure, higher delivered charge, suboptimal electrode contact, or irritant formulation conditions, a clear dose–response relationship between current density and adverse events could not be established because current density, electrode area, cumulative charge, and formulation characteristics were inconsistently reported across studies [7,8,9,10,11,12,13,14,15,16,17,18,19,20,21,22,23,24,25,26,27].

Iontophoresis produces temporary, mild skin reactions while avoiding irreversible barrier disruption, as consistently demonstrated by controlled safety studies. These reactions primarily include erythema and transient increase in transepidermal water loss [25,26]. Both electrical parameters and formulation-related factors may contribute to local tolerability, as surfactant pretreatment was associated with increased erythema and transepidermal water loss compared with iontophoresis alone [25].

These effects are reversible and do not vary considerably between different ethnic skin types. It is important to achieve a standardized modulation of the current and of the electrode design as clinical trials have reported sporadic cases of localized burns. Overall, the available evidence suggests an acceptable safety profile when appropriate protocols are used, but the occurrence of localized burns highlights the need for standardized reporting of current density, electrode area, treatment duration, cumulative charge, formulation characteristics, and device-related parameters [7,8,9,10,11,12,13,14,15,16,17,18,19,20,21,22,23,24,25,26,27].

Skin responses to histamine iontophoresis are context-dependent and influenced by physiological and environmental factors. Responses are reduced during the summer compared to winter. Female subjects show larger wheal responses than males, and depending on the anatomical region in which the procedure is done, skin reaction will differ, being stronger in proximal areas like the shoulder and weaker in distal regions such as the hands and feet [24]. When interpreting the results of dermatological interventions involving the use of iontophoresis, researchers must account for individual and environmental variability to guarantee precise diagnostic and pharmacologic analysis.

### 4.8. Comparison with Alternative Penetration-Enhancement Platforms and Translational Considerations

Compared with microneedling, iontophoresis has the advantage of being non-invasive and electrically controllable, whereas microneedles create transient microchannels that can markedly increase permeability but still require mechanical disruption of the stratum corneum. Microneedle systems have shown strong permeation-enhancing potential, including flux increases of up to four orders of magnitude for calcein and therapeutically relevant plasma levels of naltrexone after microneedle pretreatment. Iontophoresis may therefore be preferable when the target compound is charged or polar and when controlled, reversible delivery is desired without creating microchannels in the skin [2].

Recent experimental approaches combining iontophoresis with porous or dissolving microneedles suggest that these techniques may be complementary rather than mutually exclusive, with reported improvements in skin permeation, biocompatibility, and permeation flux in combined systems. Compared with phonophoresis/sonophoresis, iontophoresis allows drug delivery to be modulated by current intensity, treatment duration, and electrode surface area, whereas sonophoresis depends on ultrasound-related cavitation, thermal, convective, and mechanical effects. Sonophoresis has shown substantial enhancement of drug permeation, including a 6.6-fold increase in ketoprofen permeation compared with passive delivery, but its mechanisms remain incompletely understood, and fewer recent studies have evaluated this approach [1]. Compared with laser-assisted delivery, iontophoresis avoids ablative or thermal disruption of the skin barrier and may therefore be more suitable for repeated applications when the target compound is charged or polar [1,2]. Conversely, laser-assisted delivery can create microchannels and may be advantageous when stronger barrier disruption is required, as shown by its use in ablative fractional laser-assisted PDT protocols [16]. However, laser-based approaches may be more procedure-dependent and associated with greater downtime, whereas iontophoresis offers a non-ablative and electrically controllable alternative [1,2].

From a safety perspective, iontophoresis-related irritation is generally current-dependent and limited when low currents are used, whereas sonophoresis has also been reported to be painless and non-irritating when used within appropriate limits [2]. Thus, iontophoresis should be positioned not as a universally superior technique, but as a controllable and non-invasive platform that may be especially useful for charged or polar molecules, while microneedling and sonophoresis may offer advantages for selected molecules or settings requiring stronger barrier disruption [1].

### 4.9. Limitations of the Evidence and of the Review Process

The review process has some limitations, including the absence of a formal assessment of reporting bias and certainty of evidence, as well as the marked heterogeneity of the included studies, which limited quantitative synthesis. Many included studies were small, single-center studies, and several non-randomized studies used before-after designs, thereby increasing susceptibility to bias and imprecision. In addition, although most domain-level judgments were rated as low risk or high quality, several studies were limited by unclear methodological details and some concerns regarding randomization or intervention adherence, which further reduced the precision and generalizability of the findings. Some outcomes were reported inconsistently or without sufficient detail for standardized comparison. The interpretability of the evidence was further limited by the wide variability in follow-up duration, ranging from acute pharmacologic or physiologic assessments over minutes to hours to clinical studies with follow-up extending over weeks, months, or years. This makes short-term delivery effects difficult to compare with durable clinical outcomes and limits conclusions regarding long-term effectiveness and late safety. Although a few studies reported follow-up beyond the immediate post-treatment period, long-term follow-up was limited or inconsistently reported in most clinical trials, restricting conclusions about durability of response, recurrence, and delayed adverse events. The descriptive subgroup synthesis should also be interpreted cautiously because several potentially relevant modifiers, including formulation pH, ionic state, molecular weight, electrode area, cumulative delivered charge, and current density, were not consistently reported in the original studies. Publication bias also cannot be excluded, particularly in cosmetic and aesthetic indications, where small uncontrolled studies with favorable outcomes may be more likely to be published than negative or inconclusive studies. These limitations should be considered when interpreting the apparent clinical benefit of iontophoresis across indications.

## 5. Conclusions

Iontophoresis may be a useful non-invasive platform for enhancing topical and transdermal drug delivery in selected dermatologic conditions. Its clinical relevance seems greatest when treatment efficacy is limited by poor penetration through anatomical or physicochemical barriers, such as the stratum corneum, nail plate, or hyperkeratotic skin. However, the strength of evidence differs substantially across indications, and iontophoresis should therefore be considered a delivery-enhancement strategy rather than a broadly established stand-alone therapeutic modality.

The strongest and most consistent evidence was observed in pigmentary disorders and infectious conditions. In melasma and post-inflammatory hyperpigmentation, iontophoresis improved objective pigmentation outcomes and MASI scores when used with vitamin C or tranexamic acid [9,12,20,21,22]. In infectious diseases, iontophoretic acyclovir shortened healing time in herpes labialis, while terbinafine iontophoresis increased nail drug concentrations and improved mycological outcomes in onychomycosis [15,27]. These findings suggest that iontophoresis may be particularly useful when the target site is anatomically protected or when the delivered compound has limited passive permeability.

Evidence in inflammatory dermatoses is more heterogeneous. Psoriasis studies suggest a potential role for iontophoresis in anatomically difficult sites, such as nails and palmoplantar skin, where drug penetration is a major limitation [7,13,14]. Conversely, available data in chronic eczema did not show a clear additive benefit over comparator treatments, supporting a more cautious interpretation for this indication [23].

Preliminary signals were also reported in chronic ulcers and microvascular disorders, particularly through enhanced local perfusion and limited systemic exposure after iontophoretic delivery of vasoactive peptides and prostacyclin analogues [8,17,18,19]. However, some studies primarily assessed physiologic or safety outcomes rather than definitive healing endpoints, so the clinical impact in these indications remains uncertain.

In atrophic acne scars, iontophoretic delivery of tretinoin and estriol induced a persistent clinical improvement. These results indicate that in the setting of structural dermal damage, iontophoresis can increase intradermal bioavailability and possibly have a remodeling effect while being non-invasive [10,11]. Similarly, in photodynamic therapy, enhanced photosensitizer delivery and shorter incubation times were observed when using iontophoresis without compromising therapeutic efficacy [16].

Importantly, safety validation studies consistently showed that when proper parameters are used, iontophoresis produces solely transient erythema and reversible barrier disruption without persistent skin impairment [25,26]. The use of this approach has a favorable safety and tolerability profile, although the occasional occurrence of localized burns highlights the requirement for a standard protocol. A crucial consideration when interpreting the outcomes of iontophoresis is that they may vary according to patient-specific and environmental variables, meaning that the response to therapy delivered by iontophoresis is not uniform and can be influenced by both biological and external factors [24].

Future research should define standardized, indication-specific iontophoresis protocols, including current density, treatment duration, electrode area, polarity, formulation characteristics, and cumulative delivered charge. Benchmark regimens, such as 0.5 mA/cm^2^ for 20 min when clinically appropriate, should be prospectively tested together with lower-current protocols to identify optimal efficacy–tolerability thresholds. Multicenter randomized controlled trials enrolling more than 100 patients per indication, with follow-up beyond 12 months, are needed to assess durability of response, recurrence, patient-reported outcomes, adherence, cost-effectiveness, and delayed adverse events. Head-to-head comparisons with emerging penetration-enhancement technologies, including microneedling, laser-assisted delivery, and sonophoresis, are also required to clarify the clinical settings in which iontophoresis provides a distinct advantage.

## Data Availability

The PRISMA 2020 checklist, PRISMA flow diagram, full search strategies, and summary extraction tables are provided in the Supplementary Materials; additional review materials are available from the corresponding author upon reasonable request.

## Supplementary Materials

The following supporting information can be downloaded at https://www.mdpi.com/article/10.3390/ph19050765/s1. Table S1: PRISMA 2020 checklist for the reporting of this systematic review. Reference [28] has been cited in the Supplementary Materials.

## Abbreviations

The following abbreviations are used in this manuscript:

AE	Adverse Event
AFL-PDT	Ablative Fractional Laser Photodynamic Therapy
CENTRAL	Cochrane Central Register of Controlled Trials
CGRP	Calcitonin Gene-Related Peptide
DLQI	Dermatology Life Quality Index
ESIF	Erythema, Scaling, Induration, Fissuring
JBI	Joanna Briggs Institute
MASI	Melasma Area and Severity Index
NAPSI	Nail Psoriasis Severity Index
PDT	Photodynamic Therapy
PIH	Post-Inflammatory Hyperpigmentation
PRISMA	Preferred Reporting Items for Systematic Reviews and Meta-Analyses
PROSPERO	International Prospective Register of Systematic Reviews
PUVA	Psoralen plus Ultraviolet A
QUADAS-2	Quality Assessment of Diagnostic Accuracy Studies-2
RCTs	Randomized Clinical Trials
RoB 2	Version 2 of the Cochrane Risk-of-Bias tool
SSc-DU	Systemic Sclerosis-related Digital Ulcers
TXA	Tranexamic Acid
VIP	Vasoactive Intestinal Peptide
